# Flaky Paint Dermatosis in Kwashiorkor

**DOI:** 10.4269/ajtmh.21-0487

**Published:** 2021-10-04

**Authors:** Palaniappan Vijayasankar, Kaliaperumal Karthikeyan

**Affiliations:** Department of Dermatology, Venereology, and Leprosy, Sri Manakula Vinayagar Medical College and Hospital, Pondicherry, India

**Figure 1.**
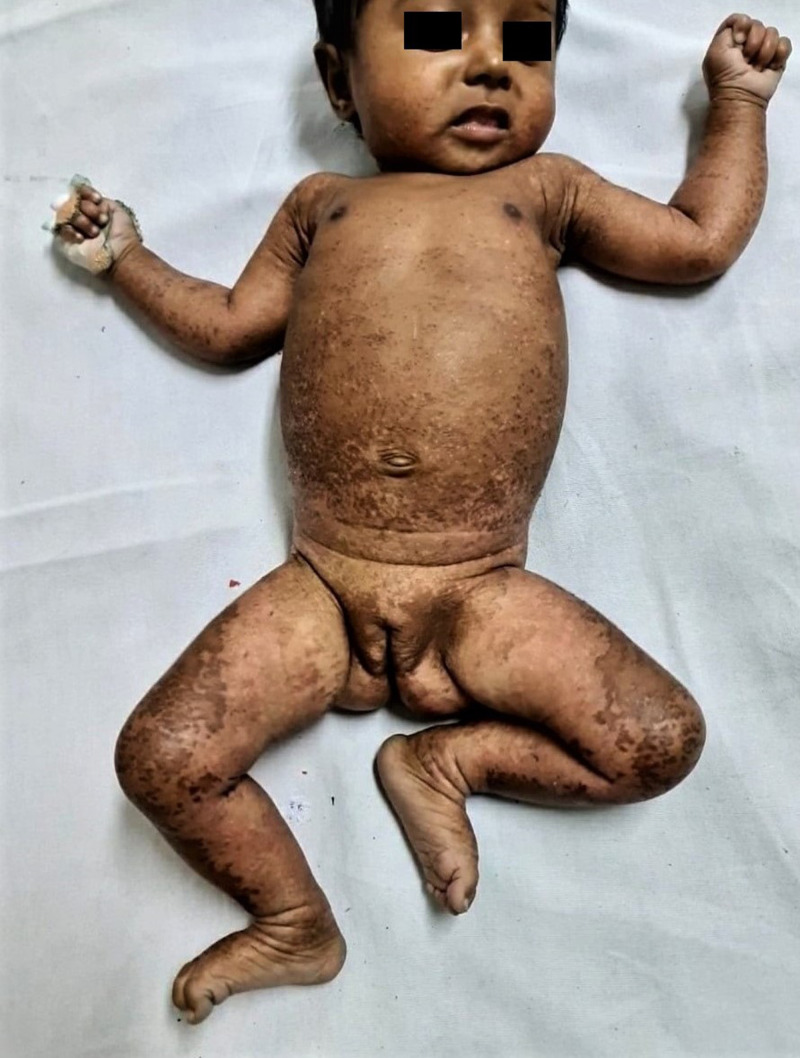
Widespread, dry, desquamative lesions characteristic of flaky paint dermatosis. This figure appears in color at www.ajtmh.org.

A 3-month-old infant was presented with a 4-week history of scaly lesions on face, trunk, and limbs. The infant’s mother indicated that the infant had a history of diarrhea for 6 weeks and failure to gain weight. On physical examination, the infant was irritable, dehydrated, and exhibited generalized edema; her weight for age was in the 64^th^ percentile. Cutaneous examination revealed dry, desquamative, hyperpigmented, patchy, and macular lesions on the face, trunk, and bilateral upper and lower limbs (Figure 1). The scalp hair was dry and brittle. Laboratory parameters showed hypoalbuminemia (1.5 g/dL), hypokalemia (2.1 mEq/L), and normal serum zinc levels. The clinical condition was characteristic of kwashiorkor with “flaky paint dermatosis.” The infant was admitted to the hospital and managed with total parenteral nutrition, topical emollients, and multivitamin supplements. The child improved clinically within 2 weeks. The mother was advised to continue exclusive breastfeeding.

Kwashiorkor, a form of severe protein-energy malnutrition, is common in areas of famine and restricted food supply that affects older infants and young children. Clinically, it is characterized by normal weight for age, irritability, anorexia, edema, distended abdomen, hepatomegaly, and dermatosis.^1^^,^^2^ The cutaneous and hair manifestations are attributable to low methionine levels that impair sulphation of the keratin.^3^

Skin manifestations of kwashiorkor progress over the course of days from dryness and atrophy to generalized hyperkeratosis and hyperpigmentation. The fragile skin peels off in an irregular manner to reveal underlying hypopigmentation (“flaky paint” or “peeling paint” dermatosis), particularly over the buttocks and limbs. The most common skin finding is the shiny, varnished-looking nature of hyperpigmentation. The hair is usually sparse, dry, and brittle, with a reddish yellow hue. The alternate bands of normal and pale hair (flag sign) reflect periods of good and poor nutrition. The nail plate changes and exhibits thinning, fissuring, ridging, or prominent koilonychia.^4^^,^^5^

The differential diagnosis includes marasmus, acrodermatitis enteropathica, free fatty acid deficiency, multiple carboxylase deficiency, pellagra (niacin deficiency), and malabsorptive syndromes. Kwashiorkor can be corrected by improving the intake of calories and protein.^2^^,^^3^ Gradual and progressive introduction of enteral feeds is the key to re-alimentation. Initially, simple sugars and fat are introduced, and proteins are started when energy is increased.^2^
